# Methylation status of the *PPP1R13L* promoter region among lung cancer patients and healthy controls. Analytical cross-sectional study

**DOI:** 10.1590/1516-3180.2018.0358230419

**Published:** 2019-08-29

**Authors:** Jiaoyang Yin, Yegang Ma, Ulla Vogel, Chunhong Wang, Ying Zhang, Huiwen Wang

**Affiliations:** I PhD. Professor, Key Laboratory of Environment and Population Health of Liaoning Education Ministry, Shenyang Medical College, Shenyang, China.; II MD. Professor/Chief Physician of Thoracic Surgery, Department of Thoracic Surgery, Liaoning Cancer Hospital, Shenyang, China.; III PhD. Professor, National Research Centre for the Working Environment, Copenhagen DK-2100, Denmark.; IV MSc. Associate Professor, Key Laboratory of Environment and Population Health of Liaoning Education Ministry, Shenyang Medical College, Shenyang, China.; V PhD. Associate Professor, Key Laboratory of Environment and Population Health of Liaoning Education Ministry, Shenyang Medical College, Shenyang, China.; VI MSc. Professor, Key Laboratory of Environment and Population Health of Liaoning Education Ministry, Shenyang Medical College, Shenyang, China.

**Keywords:** PPP1R13L protein, human [supplementary concept], Promoter region, genetic, CpG islands, Methylation, Lung neoplasms

## Abstract

**BACKGROUND:**

There is evidence that genetic predisposition and epigenetic alteration (e.g. DNA methylation) play major roles in lung cancer. In our genetic epidemiological studies, rs1970764 in oncogene *PPP1R13L* was most consistently associated with lung cancer risk. Here, we explored the role of *PPP1R13L* methylation in lung cancer development.

**DESIGN AND SETTING:**

Analytical cross-sectional study (45 lung cancer cases and 45 controls), conducted in China.

**METHODS:**

We investigated the DNA methylation status of 2,160 cytosine-phosphate-guanine (CpG) sites in the *PPP1R13L* promoter region using the EpiTYPER assay of the Sequenom MassARRAY platform.

**RESULTS:**

In the whole study group, the methylation levels of CpG-6, CpG-9, CpG-20 and CpG-21 were significantly lower and those of CpG-16 were significantly higher in cases than in controls. Among smokers, the methylation levels at five CpG sites (CpG-6, CpG-11, CpG-15, CpG-20 and CpG-21) were statistically significantly lower among cases. Among men, the methylation levels at four CpG sites (CpG-11, CpG-15, CpG-20 and CpG-21) were significantly lower among cases. Regarding smokers, the methylation levels at CpG-7.8 and CpG-21 among cases and at CpG-22 among controls were significantly lower, compared with nonsmokers. The frequency of positivity for methylation was not significantly different between lung cancer cases and controls (68.22% for cases and 71.87% for controls; P = 0.119).

**CONCLUSION:**

Our study on a Chinese population suggests that lung cancer patients have aberrant methylation status (hypomethylation tended to be more frequent) in peripheral blood leukocytes at several CpG sites in the *PPP1R13L* promoter region and that exposure to smoking may influence methylation status.

## INTRODUCTION

Lung cancer is considered to be one of the deadliest types of cancer worldwide. Lung cancer will continue to be a major health problem well through the first half of this century. It is a multifactorial disease with complex pathogenesis in which lifestyle, individual genetic background and environmental risk factors are involved. Tobacco use is an important environmental risk factor for lung cancer development. However, genetic predisposition and epigenetic alteration also play major roles in the genesis of lung cancer.^1^^,^^2^^,^^3^

The chromosomal subregion 19q13.3 harbors four genes that are involved in deoxyribonucleic acid (DNA) repair, apoptosis, ribosomal ribonucleic acid (rRNA) transcription and cell proliferation. From 3’→5’, these are: *ERCC2/XPD* [excision repair cross-complementing rodent repair deficiency, complementation group 2/xeroderma pigmentosum complementary group D]; *PPP1R13L/IASPP/RAI* [protein phosphatase 1, regulatory (inhibitor) subunit 13-like/inhibitory member of the apoptosis stimulating proteins of p53 (ASPP) family/RelA-associated inhibitor]; *CD3EAP/ASE-1* [CD3e molecule, epsilon-associated protein/antisense to ERCC1)]; and *ERCC1* [excision repair cross-complementing rodent repair deficiency, complementation group 1].^4^

DNA methylation is an epigenetic DNA modification catalyzed by DNA methyltransferase 1 (DNMT1). Gene expression can be epigenetically regulated via changes in DNA methylation. In particular, site-specific DNA methylation alterations in cytosine-phosphate-guanine (CpG) islands around the 5’-untranslated regions (5’-UTRs) of genes, including hypomethylation of oncogenes and hypermethylation of tumor suppressor genes, may be crucial promoters of cancer progression.^5^

Accumulating evidence indicates that differential DNA methylation patterns of genes are involved in lung carcinogenesis.^5^^,^^6^^,^^7^^,^^8^^,^^9^^,^^10^^,^^11^ In our genetic epidemiological fine-mapping studies, the polymorphism rs1970764 in the oncogene *PPP1R13L* has been most consistently associated with lung cancer risk.^4^ Currently, the role of *PPP1R13L* methylation in lung cancer development remains unclear.

## OBJECTIVE

To compare the DNA methylation status of the *PPP1R13L* promoter region between lung cancer patients and healthy controls, we conducted this Chinese analytical cross-sectional study.

## METHODS

### Ethical considerations

The Chinese Administration Office of Human Genetic Resources approved this protocol (no. [2001]015; approval date: May 10, 2001). All study participants granted written or oral informed consent.

### Study design and sample

No data on methylation levels in the promoter region of *PPP1R13L* have been reported in any population. We designed this cross-sectional study such that its sample consisted of 45 lung cancer cases and 45 controls. In total, 2,160 CpG sites (24 CpG sites/subject; 1,080 CpG sites for cases and 1,080 CpG sites for controls) were analyzed.

Newly diagnosed primary lung cancer cases were recruited between March 2010 and August 2012 at Liaoning Cancer Hospital, China. The diagnosis of lung cancer was based on standard clinical and histological criteria. Eligible cases had not previously undergone any treatment by means of either chemotherapy or radiotherapy. Cancer-free controls were primarily recruited from the orthopedics wards of the Affiliated Second Hospital of Shenyang Medical College, China. The controls were matched to the cases in terms of age (± 3 years), gender and ethnicity. All subjects were unrelated, and from the Han Chinese ethnic group. At enrollment, each participant was personally interviewed by doctors in order to acquire and record information on demographic characteristics, family history of cancer and lifetime history of tobacco use. Stratification was determined according to age, gender, family history of cancer, smoking history and pathological type. Family ­history and smoking status were assessed, because both of these variables are expected to be associated with *PPP1R13L* methylation.

### Determination of target region

We searched for potential CpG islands in the *PPP1R13L* promoter region in three sources: the website http://www.ncbi.nlm.nih.gov/ (GRCh38.p2 primary assembly), with the aim of intercepting sequences of 2,000 base pairs (bp) upstream and 1,000 bp downstream from the transcription start site; the website http://www.ebi.ac.uk/Tools/seqstats/emboss_cpgplot/, with the aim of predicting potential CpG islands; and the Sequenom EpiDesigner software, with the aim of designing primers for this genetic sequence.

**Figure 1.**
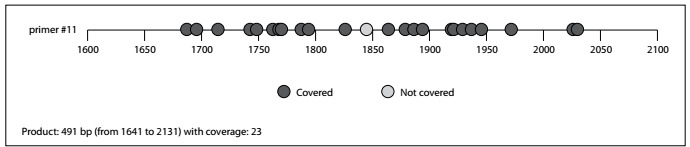
Schematic diagram of cytosine-phosphate-guanine (CpG) sites in #11 scheme of the *PPP1R13L* promoter region from CpG-1 to CpG-24 (from left to right). The sequence is from 5’ to 3’. The dots represent the CpG sites. Blue dots indicate CpG sites that could be detected. The red dot indicates a CpG site that could not be detected because of a sequencing problem.

We obtained this sequence and identified three CpG islands in the *PPP1R13L* promoter region of chr19: 45405350~45408349. Two primer schemes (#9 and #11) were recommended. We performed methylation analyses on #11 scheme region (-360 bp to +131 bp upstream and downstream from the transcription start site, chr19: 45406219-45406709) in the current study. This sequence covered a product size of 491 bp (relative sequence range: 1641-2131), from which 24 CpG sites were assessed (Figure 1).

### DNA extraction

Genomic DNA was extracted from peripheral blood samples of 1.5 ml, using the FlexiGene DNA kit 250 (Qiagen, Germany).

### DNA methylation analysis

The EpiTYPER assay on the Sequenom MassARRAY platform was used to perform the quantitative gene-specific methylation analysis. This is a tool for detection and quantitative analysis of DNA methylation using base-specific cleavage and matrix-assisted laser desorption/ionization time-of-flight mass spectrometry (MALDI-TOF MS).

In short, the steps include the following: primer design, bisulfite treatment of DNA, amplification of DNA, shrimp alkaline phosphatase (SAP) reaction, RNA transcription, base-specific cleavage using RNase A, MALDI-TOF MS analysis^12^^,^^13^ and DNA methylation standards (negative control of 0% and positive control of 100%). These were used for quality control regarding the bias of PCR amplification. The sequences of Primer-Plus-Tag (lowercase: Tag; capital: primer) were:

- LeftPrimer-PlusTag-aggaagagagGGGGTTTTTTTATTTTGGGATTTAT;
- RightPrimer-PlusTag-cagtaatacgactcactatagggagaaggctCTTATAAAACATCTAAACCTCCCCA.

### Statistical analysis

The methylation levels of CpG sites were compared between cases and controls or between stratified subgroups using Student’s t test for analyses with equal variances assumed, or the t’ test for analyses with equal variances not assumed. Differences in methylation levels of CpG according to pathological subgroup were analyzed using one-way ANOVA test or non-parametric test. The methylation frequency was compared between cases and controls using the chi-square test.

Evaluations were performed using the Statistical Package for the Social Sciences 11.0 software (SPSS Inc., Chicago, IL, USA). For all statistical analyses, dif­ferences were considered to be statistically significant if the P-value was less than 0.05.

The power of the test was calculated using online statistical software (https://www.stat.ubc.ca/~rollin/stats/ssize/n2.html). “Inference for means: comparing two independent samples” was selected. From the mean values and standard deviations that were observed for the whole study group, the chances of detecting differences in methylation levels between cases and controls at the significance level of 0.05 for CpG-6, CpG-9, CpG-16, CpG-20 and CpG-21 were 64%, 76%, 57%, 82% and 68%, respectively. Thus, larger sample sizes would be required to achieve 80% power for all these CpG sites.

## RESULTS

The study population included a total of 2,160 methylation status calls for 24 CpG sites among 90 subjects (45 lung cancer cases and 45 cancer-free controls). The basic description of the cases and controls is shown in Table 1. There were no statistically significant differences in mean age, gender or smoking history between the cases and controls. However, the cases had significantly higher frequency of family history of cancer than the controls. The distribution of pathological subtypes of the cancer cases is shown in Table 1.

**Table 1.** Basic description of lung cancer cases and controls

	Cases n (%) (n = 45)	Controls n (%) (n = 45)	P-value
Age (years)			
Mean (± SD)	58 (± 11)	60 (± 9)	0.63^a^
Gender			
Male	32 (71.1)	32 (71.1)	
Female	13 (28.9)	13 (28.9)	1.0^b^
Family history of cancer			
No	40(88.9)	45 (100.0)	
Yes	5(11.1)	0 (0.0)	0.02^b^
Smoking history			
Never	18 (40.0)	25 (55.6)	
Yes	27 (60.0)	20 (44.4)	0.14^b^
Pathological type			
Squamous carcinoma	20 (44.4)		
Adenocarcinoma	14 (31.1)		
Other	11 (24.4)		

SD = standard deviation; ^a^for t test; ^b^for χ^2^ test (two-sided).

**Figure 2.**
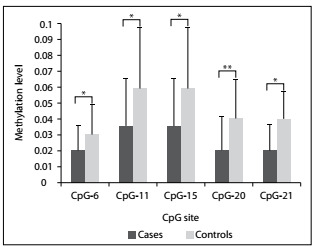
Significantly lower methylation level of the cytosine-phosphate-guanine (CpG) sites (CpG-6, CpG-11, CpG-15, CpG-20 and CpG-21) in cases than in controls, among smokers. Error bars represent standard deviation (SD). P-values were calculated using Student’s t test. *P < 0.05; **P < 0.01.

The target sequence included 24 different CpG sites, denoted CpG-1.2, CpG-3, CpG-4, CpG-5, CpG-6, CpG-7.8, CpG-9, CpG-10, CpG-11, CpG-12, CpG-13, CpG-14, CpG-15, CpG-16, CpG-17.18.19, CpG-20, CpG-21, CpG-22 and CpG-23.24 (Figure 1). The methylation status of the CpG sites in the *PPP1R13L* promoter region for the cases and controls is shown in Table 2. Some of the CpG sites were analyzed together, which resulted in reporting of mean methylation levels for CpG-1.2, CpG-7.8, CpG-17.18.19 and CpG-23.24, respectively. Equal variation was assumed for all methylation levels except for CpG-3, CpG-4, CpG-9 and CpG-16 in the whole group and for CpG-9 among nonsmokers and among women. The mean methylation levels of all CpG sites were below 10%, except for CpG-10, CpG-14, CpG-16 and CpG-22. The standard deviations were close to the mean values, thus indicating the existence of substantial inter-individual variation in the methylation levels of these CpG sites (Table 2**,**Table 3 and Figure 2).

**Table 2.** Methylation status of selected cytosine-phosphate-guanine (CpG) sites in the *PPP1R13L* promoter region, compared between cases and controls in the whole group

CpG site	Group^a^	N	Mean	Standard deviation	P-value^b^
CpG-1.2^c^	1	43	0.0586	0.05809	0.74
	2	45	0.0547	0.05392	
CpG-3	1	33	0.0752	0.11046	0.07^d^
	2	42	0.0357	0.05662	
CpG-4	1	43	0.0007	0.00258	0.13^d^
	2	43	0.0116	0.04649	
CpG-5	1	33	0.0012	0.00696	0.45
	2	35	0.0003	0.00169	
CpG-6	1	43	0.0186	0.01684	0.04
	2	44	0.0339	0.04596	
CpG-7.8^c^	1	43	0.0788	0.07089	0.70
	2	45	0.0844	0.06525	
CpG-9	1	33	0.0176	0.01838	0.036^d^
	2	32	0.0331	0.03658	
CpG-10	1	44	0.2232	0.14323	0.09
	2	45	0.1760	0.11659	
CpG-11	1	44	0.0443	0.03624	0.052
	2	45	0.0600	0.03885	
CpG-12^e^	1	0	-	-	-
	2	0	-	-	
CpG-13	1	43	0.0005	0.00305	0.72
	2	42	0.0007	0.00342	
CpG-14	1	44	0.2232	0.14323	0.09
	2	45	0.1760	0.11659	
CpG-15	1	44	0.0443	0.03624	0.052
	2	45	0.0600	0.03885	
CpG-16	1	44	0.2491	0.19263	0.036
	2	45	0.1607	0.19949	
CpG-17.18.19^c^	1	44	0.0266	0.02342	0.80
	2	45	0.0280	0.02897	
CpG-20	1	44	0.0198	0.02205	0.007
	2	45	0.0380	0.03788	
CpG-21	1	43	0.0242	0.02363	0.019
	2	44	0.0364	0.02402	
CpG-22	1	44	0.1236	0.06627	0.86
	2	45	0.1267	0.08710	
CpG-23.24^c^	1	43	0.0186	0.06760	0.69
	2	43	0.0137	0.04220	

^a^1: cases, with 1,080 CpG sites tested; 2: controls, with 1,080 CpG sites tested; ^b^for t test; ^c^mean values of these sites; ^d^for t’ test; ^e^testing failed.

**Table 3.** Methylation status of selected cytosine-phosphate-guanine (CpG) sites in the *PPP1R13L* promoter region, compared between cases and controls in different subgroups^a^

Subgroup/CpG site	Group^b^	N	Mean	Standard deviation	P-value^c^
Smokers					
CpG-6	1	26	0.02	0.016	0.047
	2	20	0.03	0.019	
CpG-11	1	27	0.0356	0.03030	0.024
	2	20	0.0590	0.03865	
CpG-15	1	27	0.0356	0.03030	0.024
	2	20	0.0590	0.03865	
CpG-20	1	27	0.02	0.022	0.005
	2	20	0.04	0.025	
CpG-21	1	26	0.02	0.017	< 0.0001
	2	19	0.04	0.017	
Nonsmokers					
CpG-9	1	14	0.0207	0.01940	0.039^d^
	2	17	0.10435	0.03757	
CpG-3	1	13	0.0946	0.10047	0.045
	2	13	0.0365	0.06733	
Men					
CpG-11	1	31	0.0403	0.03027	0.047
	2	32	0.0569	0.03421	
CpG-15	1	31	0.0403	0.03027	0.047
	2	32	0.0569	0.03421	
CpG-20	1	31	0.0197	0.02359	0.005
	2	32	0.0375	0.02476	
CpG-21	1	30	0.0230	0.02246	0.028
	2	31	0.0358	0.02187	
CpG-16	1	31	0.2619	0.20988	0.044
	2	32	0.1572	0.19525	
Women					
CpG-9	1	11	0 .0164	0.01433	0.007^d^
	2	9	0.0533	0.03041	

^a^Only statistically significant results are listed; ^b^1: cases, with 1,080 CpG sites tested; 2: controls, with 1,080 CpG sites tested; ^c^for t test; ^d^for t’ test.

In the whole study group, the methylation levels of CpG-6, CpG-9, CpG-20 and CpG-21 were statistically significantly lower among cases than among controls. The methylation level of CpG-16 was statistically significantly higher among cases than among controls. There were no statistically significant differences between cases and controls regarding methylation levels at all CpG sites taken together, or at the remaining CpG sites (Table 2).

Stratified analyses were made for subgroups based on smoking history, gender and pathological type. Among smokers, five CpG sites had statistically significantly lower methylation levels among cases than among controls. These were CpG-6, CpG-11, CpG-15, CpG-20 and CpG-21 (Table 3 and Figure 2). Among nonsmokers, there was statistically significantly lower methylation levels for CpG-9 and higher methylation levels for CpG-3 among cases than among controls (Table 3). Among men, lower methylation levels at four CpG sites (CpG-11, CpG-15, CpG-20 and CpG-21) were found among cases than among controls. Higher methylation levels at the CpG-16 site were found among male cases than among male controls (Table 3). Among women, only CpG-9 had significantly lower methylation levels among cases than among controls (Table 3). In addition, smokers had statistically significantly lower methylation levels at CpG-7.8 (0.0570 ± 0.0458 versus 0.1028 ± 0.0926; P = 0.033) and CpG-21 (0.0167 ± 0.0166 versus 0.0328 ± 0.0293; P for t’ test = 0.045) among cases and at CpG-22 (0.0915 ± 0.0809 versus 0.1548 ± 0.0829; P = 0.014) among controls, compared with nonsmokers. The methylation level of all CpG sites taken together, or for the remaining CpG sites, did not differ signifi­cantly between cases and controls based on smoking status or gender (data not shown). Methylation levels were unaffected by pathological subgroup (data not shown).

The frequency of methylation-positive CpG sites in the *PPP1R13L* promoter region was not statistically significantly different between lung cancer cases and controls [68.22% for cases (positive versus negative = 513:239) and 71.87% for controls (positive versus negative = 557:218); χ^2^ = 2.483; P = 0.119]. Hypomethylation was most frequently observed, and was present at 81% of the aberrantly methylated CpG sites.

## DISCUSSION

### Main findings of the study

To the best of our knowledge, this is the first study investigating the association between DNA methylation in the promoter region of the oncogene *PPP1R13L* and lung cancer. We report that in the *PPP1R13L* promoter region, the methylation levels of some CpG sites in peripheral blood leukocytes differed between lung cancer cases and controls and that hypomethylation was observed more often in lung cancer cases than in controls. Lowered methylation levels were also more prominent among smokers and among men.

### Studies addressing methylation in lung cancer

The association between the methylation status of specific genes in peripheral blood and lung cancer has previously been addressed.^5^^,^^6^^,^^7^^,^^8^^,^^9^^,^^10^^,^^11^^,^^14^

In a study on the EPIC-Italy cohort and the MCCS cohort, hypomethylation of six CpG sites was associated with smoking and lung cancer risk: cg05575921 in the AHRR (aryl-hydrocarbon receptor repressor) gene; cg03636183 in the F2RL3 (F2R-like thrombin/trypsin receptor 3) gene; cg21566642 and cg05951221 in 2q37.1; cg06126421 in 6p21.33; and cg23387569 in 12q14.1. This provided evidence that smoking and possibly other factors may lead to DNA methylation changes that can be detected in peripheral blood and that are predictive of lung cancer risk.^6^

In a Chinese case-control study, it was reported that subjects with hypermethylation of the promoter region of hOGG1 (human 8-oxoguanine DNA glycosylase) had a 2.25-fold increased risk of developing non-small cell lung cancer (NSCLC), compared with methylation-free subjects.^5^ In another Chinese case-control study, it was reported that there were statistically significant differences in the promoter methylation levels of p16 (*CDKN2A*, cyclin dependent kinase inhibitor 2A), RASSF1A (Ras association domain family member 1A), and FHIT (fragile histidine triad) between lung cancer cases and controls. Moreover, it was reported that high methylation levels of the p16, RASSF1A and FHIT genes were associated with significantly increased risk of lung cancer. It was concluded that further investigation of the potential usefulness of methylation status of these three genes in clinical practice was warranted.^7^

In an American prospective nested case-control study, it was reported that hypomethylation of the p53 (tumor protein p53) gene in exons 5-8, the hypermutable region, was associated with a 2-fold increased risk of lung cancer [OR (95% CI) = 2.20 (1.04-4.65)]. It was suggested that hypomethylation status within exons 5-8 of p53 from peripheral lymphocyte DNA might be a relevant predictor of lung cancer among male smokers.^8^

A Thai case-control study found that the methylation percentage of LINE-1 (interspersed DNA repetitive sequences) loci ((u)C(u)C) was significantly higher in lung cancer patients than in healthy controls and suggested that changes in the levels and patterns of genome-wide methylation of peripheral blood mononuclear cells (PBMCs) were associated with lung cancer risk.^9^ The study confirmed that methylation of the SHOX2 (short stature homeobox gene 2) gene may be a reliable marker for lung malignancies and suggested that SHOX2 methylation in blood plasma may represent an alternative diagnostic test for patients who are unable to undergo bronchoscopy.^10^ SHOX2 encodes a homeo-domain transcription factor which has been identified as a close homologue of the SHOX gene, and both genes are involved in skeletogenesis and heart development.^10^

In a Polish case-control study, it was reported that the methylation levels of DNA from peripheral blood plasma were slightly higher in patients with small-cell lung cancer (SCLC) (75% of SCLC patients), compared with healthy individuals. The median overall survival of patients with *DCLK1* (doublecortin-like kinase 1) promoter methylation was lower than that of patients without *DCLK1* gene methylation (hazard ratio, HR = 4.235).^11^

A meta-analysis on genome-wide DNA methylation showed that smoking left a long-term signature in relation to DNA methylation and that this was a potential mechanism through which tobacco exposure would predispose towards adverse health outcomes, such as cancer, osteoporosis and lung and cardiovascular disorders.^15^ Cigarette smoking has a broad impact on genome-wide methylation that, at many loci, persists many years after smoking cessation.^15^

A multicenter case-control study that used data from six countries in Central and Eastern Europe suggested that global blood DNA methylation in peripheral blood was not associated with the risk of lung cancer among nonsmoking women.^14^

### Implications, strengths and limitations

The finding from the present study is consistent with previous observations that hypomethylation of the oncogene promoter may be an early and frequent molecular event in occurrences of cancer.^5^ However, we were unable to determine whether the changes in methylation levels were a cause or a consequence of lung cancer. The difference in methylation levels between smokers and nonsmokers among both cases and controls suggested that smoking might lead to lower methylation levels at CpG sites of the *PPP1R13L* promoter region in peripheral blood leukocytes from lung cancer patients. This would thus also provide evidence that DNA methylation may be an intermediate step between smoking and lung cancer.^15^ Altered levels of DNA methylation in the *PPP1R13L* promoter therefore might be useful in detection of lung cancer.

Case-control studies, in which methylation levels in DNA isolated from peripheral blood cells from cancer patients are compared with methylation levels in healthy controls, cannot be used to establish causal relationships and, thus, establish whether the changes in promoter methylation levels are part of the carcinogenesis or are caused by disease. Nor can cross-sectional studies be used to infer the causal relationships between exposures and outcomes. However, changes in methylation levels may be used as diagnostic or prognostic biomarkers. Prospective studies in which the biological samples are collected prior to making a diagnosis of cancer may provide evidence of causality.

*PPP1R13L* (*iASPP*) is one of three members of the ASPP (apoptosis-stimulating proteins of *p53*) family. *p53*, the guardian of the genome, plays a critical role in the induction of apoptosis, typically in response to DNA damage. The ASPP family is involved in regulation of *p53*. The ASPP family includes *ASPP1*, *ASPP2* and *ASPP* (*iASPP*). *ASPP1* and *ASPP2* are tumor suppressors, whereas the inhibitor of ASPP (*iASPP*) functions as an oncogene. *ASPP1* and *ASPP2* promote apoptosis, while overexpression of *iASPP* inhibits apoptotic cell death typically after DNA damage. In cancer cells, increased expression of *iASPP* is associated with worse prognosis.^16^ Mechanistically, changes to DNA methylation status are thought to alter the transcriptional regulation of gene expression.^13^ On the other hand, *iASPP* mRNA levels were not found to be associated with lung cancer risk in a prospective study.^17^

The current study has some limitations. Its statistical power was limited due to the relatively low number of participants. The retrospective or cross-sectional design does not allow us to determine whether the observed associations between changes in methylation status and lung cancer are causal. The matching between cases and controls regarding age, gender and ethnicity in this study was insufficient to exclude potential confounding factors such as smoking.

## CONCLUSION

Our study suggests that lung cancer patients have aberrant methylation status (hypomethylation tended to be more frequent) in peripheral blood leukocytes, for several CpG sites in the *PPP1R13L* promoter region, and that exposure to smoking may influence methylation status in this Chinese population. Further research using larger sample sizes and a prospective study design is necessary in order to assess the potentially causal roles of these changes to methylation levels at CpG sites in the *PPP1R13L* promoter region, relating to lung cancer.
